# An Omission

**Published:** 1875-05

**Authors:** 


An Omission.—There is a grievous error extant in New York and in all large cities, which we hope to see reformed. We allude to the failure on the part of the officers of the various churches to have the name of church and pastor placed conspicuously upon the structure, near the entrance. Hundreds of friends from a distance have told us of their misery on sallying forth to find a particular place of worship. We saw a stranger the other day, just opposite Grace Church, on Broadway, who, after gazing at the grand edifice for a time, approached a gentleman and asked him its name. It so happened that the person addressed was also ignorant of the name, and directed him to a sign on the side of the building. The sign read—"I. H. Brown, Sexton and Undertaker, 216 East 21st street." As the party inquiring was not at that moment in need of an undertaker, the sign failed to afford him the information sought.

Now, has it ever occurred to pastors and officers of churches, that it would be a good thing to make it reasonably *possible* for strangers to attend a particular church, without subjecting them to the necessity of accosting a considerable number of pedestrians while searching for the desired spot ? Is it not better to hang out a neat sign, than it is to compel strangers to enter a church before finding out whether it is the one sought ? Would any business man take his chances of custom, while having nothing to identify him from his numerous competitors in the same line ?

We are glad to observe that some of the churches are moving in this matter, and hope to see church-signs become universal in all large cities. The Broadway Tabernacle, corner of Broadway and 34th street, displays a tasteful shield-shaped sign, giving the name of the church as well as the name of the pastor. The society of Rev. O. B. Frothingham, worshipping in Masonic Hall, also displays a sign, giving not only the name of the society and pastor, but also the hours at which the various services are held. This is a great convenience, and we can only add one further suggestion, namely, that on all such signs the residence of the pastor be also given, in order that those who may desire to summon or consult him, can readily gain the information necessary to that end. The custom of displaying the name of the sexton and undertaker is universal among us; let us ask ourselves if it is not barely possible that some further information can be afforded, which will be quite as useful and as widely appreciated.

